# Supplementary material for: Global risk pooling mitigates financial risk from drought in hydropower-dependent countries
Source: Nat Commun. 2026 Jan 16;17:723. doi: 10.1038/s41467-025-67082-z (PMC12820157; doi:10.1038/s41467-025-67082-z)
Supplement: Supplementary file 1 — Supplementary Information [file 41467_2025_67082_MOESM1_ESM.pdf]

Supplementary Information for Global Risk Pooling Mitigates Financial Risk from Drought in Hydropower-Dependent Countries

Rosa Isabella Cuppari[1,2*], Tamlin M. Pavelsky[3] and Gregory W. Characklis[1,2]

*Corresponding author(s). E-mail(s): rosa.cuppari@gmail.com;

1 Additional Statistical Tests

Indices were developed using a criteria of $r^2 \geq 0.35$ in the training and testing datasets as well as overall (see Table 1). Additional metrics were used to evaluate normality, heteroskedasticity, and accuracy (Table 2). Most countries' generation data shows evidence of normality, though some fail one out of three tests (Shapiro-Wilk, D'Agostino-Pearson, and Anderson-Darling). While this adds uncertainty to the analysis, we do not believe it alters the conclusion that bundling the risk faced by hydropower-dependent countries can lead to reduced cost of risk management.

2 Datasets

We use remote sensing-based datasets for precipitation, snow cover extent, enhanced and normalized vegetation index, and land surface temperature. Remotely sensed data are consistently available, both temporally and spatially, between 2000 and 2022. Hydrometeorological data are complemented by data on the levelized cost of electricity (LCOE), hydrologic boundaries, and global dam locations and sizes.

The Integrated Multi-satellitE Retrievals for GPM (IMERG) algorithm integrates precipitation data collected from passive microwave estimates, microwave-calibrated infrared estimates, and precipitation gauges [1]. Its particular focus is integrating data collected by the Tropical Rainfall Measuring Mission precipitation (1998-2014) and the Global Precipitation Measurement mission (2014-present). Data are intercalibrated to the Combined Radar-Radiometer Algorithm (CORRA) and Global Precipitation Climatology Project on a rolling basis to generate a continuous precipitation time series. This analysis utilizes the "Final" monthly 0.1 x 0.1 degree product, version 06, which is aggregated to a monthly time step from the half-hourly data collected [2].

Though the IMERG precipitation dataset includes snowfall, snow cover extent can provide a a complementary measure of snowmelt magnitude and timing, which can have a substantial influence on streamflow. Data from the Terra Moderate Resolution Imaging Spectroradiometer (MODIS) provide information at the 0.05 x 0.05 degree spatial scale. The MODIS snow cover extent dataset (MOD10CM) [3] provides the Normalized Difference Snow Index (NDSI) value, the normalized difference of green and shortwave infrared wavelengths used to capture the relative percentage of high reflectance values typical of snow in visible bands. Snow cover extent, reported from 2000 to 2024, is aggregated to the monthly level using observations taken every 1-2 days. In the MOD10CM dataset, observations with some amount of snow cover that are partially obscured by clouds are assumed to underestimate total snow cover and are adjusted according to the Clear Index, which represents the portion of a grid cell obscured by cloud cover.

The above hydrometeorological data is aggregated temporally and spatially to provide possible inputs to an index. Temporally, data is averaged over the monthly and seasonal timescales. Use of the maximum and minimum values for each variable were also tested, but use of these variables provided no improvement on the indices. The data is then spatially aggregated in two ways: using country borders and subbasin boundaries. The HydroBASINS dataset developed by Lehner and Grill [4] identifies different sizes of subbasins using the location where two river branches meet, given a minimum upstream area of 100 km^2. Subbasins are categorized into 12 levels according to the Pfafstetter system, ranging from the continental to local watershed levels. The single level 4 basin within each country with the largest concentration of hydroelectric dams is identified in order to spatially aggregate environmental condition inputs (see Figure 1). The Global Reservoir and Dam Database (GRanD) is used for this purpose [5]. It includes records on nearly 7,000 reservoirs and their dams, as well as information on their purposes. Only dams whose primary purpose is hydropower generation are included in the analysis. Once the level 4 subbasin of interest is identified, available, pixel-level hydrometeorological data (i.e., temperature, precipitation, snow cover, and vegetation indices) is averaged across the entire subbasin. The variables are averaged

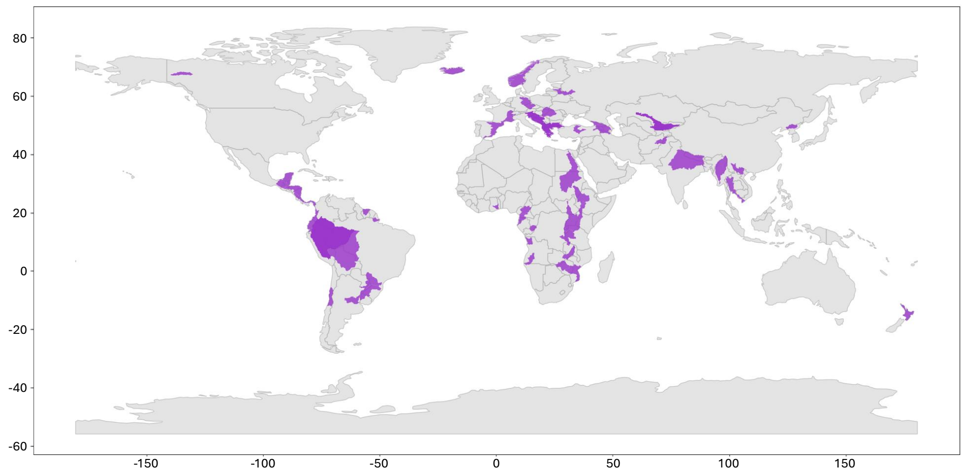

Supplementary Fig. 1: Level 4 subbasins used to spatially aggregate remotely sensed data. Basins highlighted in purple.

within country borders as well, providing a single average hydrometeorological value at two spatial scales and two temporal scales.

In summary, the indices were designed using the following steps:

1. Calculate the annual capacity factor for each hydropower-dependent country
2. Extract the average precipitation, land surface temperature, snow cover extent, and vegetation indices across two spatially bounded areas at the temporal level of the data (monthly). Data was not additionally filtered after download.
    - Within country (political borders)
    - Within the boundaries of the Level 4 subbasin in which the majority of a country's dams reside
3. Temporally aggregate the spatially aggregated data in two ways:
    - Aggregate at the seasonal level (i.e., winter, spring, summer, fall)
    - Take the annual average of the spatially aggregated data
4. Evaluate the accuracy of a linear regression using any combination of variables. A training/test set split of 80/20 was used. This process was done automatically using a Python script that saved the combination meeting the test criteria of ≥ 0.35 r^2 and less than four statistically significant input variables (two-tailed p-value ≤ 0.05).
5. Manually verify the accuracy of each index

3 Shapley Values

There exists a rich literature on capital allocation in the context of insurance, used to price premiums for individual lines of business within a portfolio [6–9], comparable to policies for individual countries within a pool. The objective of the literature, which draws from the fields of game theory [6, 10, 11], finance [7], and optimization methods, is to allocate risk capital with fairness, such that the risk capital or premium associated with an individual participant or business line is reflective of the individual's marginal contribution to the overall risk taken on by an insurer.

Advances in game theory by Lloyd Shapley set the foundation for the eponymous Shapley method and value, a game theoretic approach to allocating the marginal gains or costs attributable to an individual member of a coalition [12]. It considers all possible coalitions, or subsets of pool participants, when calculating the marginal contribution of an individual participant to the coalition's losses (or gains). It is also considered a coherent risk allocation method, i.e., one that meets principles consistent with economic 'fairness' (subadditivity, monotonicity, homogeneity, and translation invariance), as discussed in Denault [6].

Supplementary References

[1] Huffman, G.J., Bolvin, D.T., Braithwaite, D., Hsu, K.-L., Joyce, R.J., Kidd, C., Nelkin, E.J., Sorooshian, S., Stocker, E.F., Tan, J., Wolff, D.B., Xie, P.: In: Levizzani, V., Kidd, C., Kirschbaum, D.B., Kummerow, C.D., Nakamura, K., Turk, F.J. (eds.) Integrated Multi-satellite Retrievals for the Global Precipitation Measurement (GPM) Mission (IMERG). Advances in global change research, vol. 67, pp. 343–353. Springer, Cham (2020). https://doi.org/10.1007/978-3-030-24568-9_19

[2] Huffman, G.J., Stocker, E.F., Bolton, D.T., Nelkin, E.J., Tan, J.: Gpm imerg final precipitation l3 1 month 0.1 degree x 0.1 degree v06. NASA Goddard Earth Sciences Data and Information Services Center (2019) https://doi.org/10.5067/gpm/imerg/3b-month/06

[3] Hall, D.K., Riggs, G.A.: Modis/terra snow cover monthly l3 global 0.05deg cmg, version 61. NASA National Snow and Ice Data Center Distributed Active Archive Center (2021) https://doi.org/10.5067/modis/mod10cm.061

[4] Lehner, B., Grill, G.: Global river hydrography and network routing: baseline data and new approaches to study the world's large river systems. Hydrological processes **27**(15), 2171–2186 (2013) https://doi.org/10.1002/hyp.9740

[5] Lehner, B., Liermann, C.R., Revenga, C., Vörösmarty, C., Fekete, B., Crouzet, P., Döll, P., Endejan, M., Frenken, K., Magome, J., Nilsson, C., Robertson, J.C., Rödel, R., Sindorf, N., Wisser, D.: High-resolution mapping of the world's reservoirs and dams for sustainable river-flow management. Frontiers in Ecology and the Environment **9**(9), 494–502 (2011) https://doi.org/10.1890/100125

[6] Denault, M.: Coherent Allocation of Risk Capital (2001). https://www.ressources-actuarielles.net/EXT/ISFA/1226.nsf/0/e0678cad2d06cd0bc1257bd20041fcd1/$FILE/CoherentAllocation.pdf

[7] Myers, S.C., Read, J.A.: Capital allocation for insurance companies. The Journal of Risk and INsurance **68**(4), 545–580 (2001) https://doi.org/10.2307/2691539

[8] Venter, G.G.: Capital allocation survey with commentary. North American Actuarial Journal **8**(2), 96–107 (2004) https://doi.org/10.1080/10920277.2004.10596139

[9] Zanjani, G.: Pricing and capital allocation in catastrophe insurance. Journal of financial economics **65**(2), 283–305 (2002) https://doi.org/10.1016/S0304-405X(02)00141-1

[10] Powers, M.R.: Using Aumann-Shapley Values to Allocate Insurance Risk: The Case of Inhomogenous Losses (2007). https://www.planchet.net/EXT/ISFA/1226.nsf/0/cb3a65c71d6a2e73c1257a7c00682a43/$FILE/naaj0703-7.pdf

[11] Tsanakas, A., Barnett, C.: Risk capital allocation and cooperative pricing of insurance liabilities. Insurance: Mathematics and Economics **33**(2), 239–254 (2003) https://doi.org/10.1016/S0167-6687(03)00137-9

[12] Shapley, L.S.: A value for n-person games, Tucker and luce edn. AM-28, vol. II, pp. 307–317. Princeton University Press, Princeton, New Jersey (1953). https://www.jstor.org/stable/j.ctt1b9x1zv

Supplementary Table 1: Country-level index components with corresponding two-tailed p-values relative to a t-test. Constants omitted.

Country	Inputs with Coefficients	95% Confidence Intervals	Two-tailed p-values
Albania	($-3.45 * 10^{-4}$)(summer_LST)	$-6.14 * 10^{-4}$, $-7.65 * 10^{-5}$	0.02
	(1.69)(fall_precip)	0.50, 2.88	0.01
Austria	($1.35 * 10^{-4}$)(summer_EVI)	$-1.54 * 10^{-6}$, $2.71 * 10^{-4}$	0.05
	($-2.40 * 10^{-4}$)(LST)	$-4.63 * 10^{-4}$, $-1.76 * 10^{-5}$	0.04
Chile	(1.72)(precip_4)	0.77, 2.67	0.00
	($-7.86 * 10^{-3}$)(snow_4)	$-1.39 * 10^{-2}$, $-1.82 * 10^{-3}$	0.01
	($1.82 * 10^{-3}$)(snow)	$8.59 * 10^{-4}$, $2.78 * 10^{-3}$	0.00
Costa Rica	($-4.62 * 10^{-4}$)(summer_LST_4)	$-7.04 * 10^{-4}$, $-2.20 * 10^{-4}$	0.00
	($-1.45 * 10^{-4}$)(summer_EVI_4)	$-2.19 * 10^{-4}$, $-7.13 * 10^{-5}$	0.00
Croatia	(1.46)(precip)	0.65, 2.27	0.00
	($1.71 * 10^{-4}$)(fall_EVI)	$7.11 * 10^{-5}$, $2.71 * 10^{-4}$	0.00
Ecuador	($1.45 * 10^{-4}$)(fall_LST)	$3.43 * 10^{-5}$, $2.55 * 10^{-4}$	0.01
	($-2.29 * 10^{-4}$)(fall_LST_4)	$-3.89 * 10^{-4}$, $-6.79 * 10^{-5}$	0.01
Georgia	($1.43 * 10^{-4}$)(LST_4)	$2.64 * 10^{-5}$, $2.60 * 10^{-4}$	0.02
	($2.28 * 10^{-4}$)(EVI_4)	$7.00 * 10^{-5}$, $3.86 * 10^{-4}$	0.01
Latvia	($-4.75 * 10^{-4}$)(LST)	$-7.38 * 10^{-4}$, $-2.12 * 10^{-4}$	0.00
Norway	($5.42 * 10^{-4}$)(LST_4)	$9.22 * 10^{-5}$, $9.92 * 10^{-4}$	0.02
	($-5.74 * 10^{-4}$)(LST)	$-9.43 * 10^{-4}$, $-2.05 * 10^{-4}$	0.00
Pakistan	(3.31)(summer_precip_4)	1.65, 4.97	0.00
	($2.88 * 10^{-4}$)(LST)	$3.67 * 10^{-5}$, $5.4 * 10^{-4}$	0.03
Panama	($-9.12 * 10^{-4}$)(LST_4)	$-1.48 * 10^{-3}$, $3.45 * 10^{-4}$	0.00
Paraguay	(1.21)(fall_precip)	0.28, 2.14	0.01
	($-3.34 * 10^{-4}$)(summer_LST_4)	$-5.88 * 10^{-4}$, $-7.94 * 10^{-5}$	0.01
Slovenia	($4.95 * 10^{-3}$)(summer_snow_4)	$1.95 * 10^{-3}$, $7.95 * 10^{-3}$	0.00
	(0.99)(summer_precip_4)	0.26, 1.72	0.01
Uruguay	($-1.83 * 10^{-3}$)(LST)	$-2.88 * 10^{-3}$, $-7.85 * 10^{-4}$	0.00
	($-2.09 * 10^{-4}$)(fall_EVI)	$-3.90 * 10^{-4}$, $-2.71 * 10^{-5}$	0.03
Zambia	(0.70)(precip_4)	0.07, 1.34	0.03
	($6.77 * 10^{-4}$)(spring_LST_4)	$3.1 * 10^{-4}$, $1.04 * 10^{-3}$	0.00
	($-6.53 * 10^{-4}$)(LST_4)	$-1.05 * 10^{-3}$, $-2.54 * 10^{-4}$	0.00

Supplementary Table 2: Metrics evaluating hydropower capacity factor data normality and hetereoscedasticity as well as index performance. The Anderson-Darling test is evaluated with respect to p = 0.01

Country	White Test	Breusch-Pagan Test	Shapiro-Wilk Test	D'Agostino-Pearson Test	Anderson-Darling Test	MAE	Normalized Nash-Sutcliffe Efficiency
Albania	0.07	0.56	0.44	0.59	normal	0.06	0.62
Austria	0.46	0.24	0.97	0.95	normal	0.03	0.64
Chile	0.38	0.45	0.09	0.32	normal	0.05	0.74
Costa Rica	0.67	0.62	0.19	0.06	normal	0.03	0.66
Croatia	0.19	0.40	0.28	0.43	normal	0.05	0.73
Ecuador	0.40	0.81	0.51	0.40	normal	0.04	0.71
Georgia	0.53	0.30	0.87	0.88	normal	0.02	0.68
Latvia	0.71	0.80	0.73	0.55	normal	0.03	0.62
Norway	0.64	0.23	0.37	0.27	normal	0.02	0.63
Pakistan	0.46	0.63	0.04	0.26	normal	0.05	0.57
Panama	0.26	0.54	0.04	0.21	normal	0.04	0.63
Paraguay	0.31	0.42	0.05	0.18	normal	0.05	0.70
Slovenia	0.32	0.20	0.30	0.12	normal	0.04	0.67
Uruguay	0.69	0.32	0.22	0.17	normal	0.11	0.62
Zambia	0.21	0.35	0.88	0.95	normal	0.04	0.65
