# Supplementary material for: Global risk pooling mitigates financial risk from drought in hydropower-dependent countries
Source: Nat Commun. 2026 Jan 16;17:723. doi: 10.1038/s41467-025-67082-z (PMC12820157; doi:10.1038/s41467-025-67082-z)
Supplement: Supplementary file 2 — Reporting Summary [file 41467_2025_67082_MOESM2_ESM.pdf]

nature portfolio

Corresponding author(s): Rosa Isabella Cuppari

Last updated by author(s): Oct 29, 2025

Reporting Summary

Nature Portfolio wishes to improve the reproducibility of the work that we publish. This form provides structure for consistency and transparency in reporting. For further information on Nature Portfolio policies, see our Editorial Policies and the Editorial Policy Checklist.

Statistics

For all statistical analyses, confirm that the following items are present in the figure legend, table legend, main text, or Methods section.

n/a	Confirmed	
[ ]	[x]	The exact sample size (*n*) for each experimental group/condition, given as a discrete number and unit of measurement
[x]	[ ]	A statement on whether measurements were taken from distinct samples or whether the same sample was measured repeatedly
[ ]	[x]	The statistical test(s) used AND whether they are one- or two-sided<br>*Only common tests should be described solely by name; describe more complex techniques in the Methods section.*
[x]	[ ]	A description of all covariates tested
[ ]	[x]	A description of any assumptions or corrections, such as tests of normality and adjustment for multiple comparisons
[ ]	[x]	A full description of the statistical parameters including central tendency (e.g. means) or other basic estimates (e.g. regression coefficient) AND variation (e.g. standard deviation) or associated estimates of uncertainty (e.g. confidence intervals)
[ ]	[x]	For null hypothesis testing, the test statistic (e.g. *F*, *t*, *r*) with confidence intervals, effect sizes, degrees of freedom and *P* value noted<br>*Give P values as exact values whenever suitable.*
[x]	[ ]	For Bayesian analysis, information on the choice of priors and Markov chain Monte Carlo settings
[x]	[ ]	For hierarchical and complex designs, identification of the appropriate level for tests and full reporting of outcomes
[x]	[ ]	Estimates of effect sizes (e.g. Cohen's *d*, Pearson's *r*), indicating how they were calculated

Our web collection on statistics for biologists contains articles on many of the points above.

Software and code

Policy information about availability of computer code

Data collection	n/a
Data analysis	Code used for data analysis available at https://github.com/rcuppari/Hydro_Risk_Pooling. It is linked from the Zenodo repository (http://doi.org/10.5281/zenodo.17329375). Analysis was undertaken using Python 3.7 using the geopandas (version 0.10.2), shapely (version 1.8.0), statsmodels (0.13.2), scikit-learn (version 1.0.2), and scipy (version 1.7.1) packages.

For manuscripts utilizing custom algorithms or software that are central to the research but not yet described in published literature, software must be made available to editors and reviewers. We strongly encourage code deposition in a community repository (e.g. GitHub). See the Nature Portfolio guidelines for submitting code & software for further information.

Data

Policy information about availability of data

All manuscripts must include a data availability statement. This statement should provide the following information, where applicable:

- Accession codes, unique identifiers, or web links for publicly available datasets
- A description of any restrictions on data availability
- For clinical datasets or third party data, please ensure that the statement adheres to our policy

Data on index insurance contract formulation and costs of risk management generated in this study have been deposited in a Zenodo repository (DOI: 10.5281/zenodo.17329375). Input data used in this analysis is publicly available and can be found on the Earthdata database: land surface temperature (DOI: 10.5067/modis/mod11c3.061), precipitation (DOI: 10.5067/gpm/imerg/3b-month/06), vegetation indices (DOI: 10.5067/modis/mod13c2.061), and snow cover extent (DOI:

10.5067/modis/mod10cm.061). Basin boundary data is taken from the HYBAS database and the Global Reservoir and Dam database provided coordinates and uses for dams (DOI: 10.1890/100125). Additional data used in this study can be found in the Zenodo repository (DOI: 10.5281/zenodo.17329375).

Research involving human participants, their data, or biological material

Policy information about studies with human participants or human data. See also policy information about sex, gender (identity/presentation), and sexual orientation and race, ethnicity and racism.

Reporting on sex and gender	n/a
Reporting on race, ethnicity, or other socially relevant groupings	n/a
Population characteristics	n/a
Recruitment	n/a
Ethics oversight	n/a

Note that full information on the approval of the study protocol must also be provided in the manuscript.

Field-specific reporting

Please select the one below that is the best fit for your research. If you are not sure, read the appropriate sections before making your selection.

☐ Life sciences ☐ Behavioural & social sciences ☒ Ecological, evolutionary & environmental sciences

For a reference copy of the document with all sections, see nature.com/documents/nr-reporting-summary-flat.pdf

Ecological, evolutionary & environmental sciences study design

All studies must disclose on these points even when the disclosure is negative.

Study description	This study uses remotely sensed data on land surface temperature, vegetation indices, precipitation, and snow cover extent to design indices representing hydropower generation in hydropower-reliant countries. The indices are used as the basis of index insurance contracts, which are priced when considered individually and pooled. The cost of insurance is compared to the opportunity cost of holding reserves for an equivalent level of payouts.
Research sample	All countries relying on hydropower for at least 25% of their domestic electricity generation were included.
Sampling strategy	All countries relying on hydropower for at least 25% of their domestic electricity generation were included.
Data collection	No data was collected as part of this study. All data is publicly available on the Earthdata, HYBAS, and Global Reservoir and Dam databases.
Timing and spatial scale	Monthly data from 2002 to 2023 is used, collected at the 1 km x 1 km, 0.05 deg x 0.05 deg, and 0.1 deg x 0.1 deg spatial scales.
Data exclusions	n/a
Reproducibility	All data and code are publicly available.
Randomization	n/a
Blinding	n/a

Did the study involve field work? ☐ Yes ☒ No

Reporting for specific materials, systems and methods

We require information from authors about some types of materials, experimental systems and methods used in many studies. Here, indicate whether each material, system or method listed is relevant to your study. If you are not sure if a list item applies to your research, read the appropriate section before selecting a response.

Materials & experimental systems

n/a	Involved in the study
☒	☐ Antibodies
☒	☐ Eukaryotic cell lines
☒	☐ Palaeontology and archaeology
☒	☐ Animals and other organisms
☒	☐ Clinical data
☒	☐ Dual use research of concern
☒	☐ Plants

Methods

n/a	Involved in the study
☒	☐ ChIP-seq
☒	☐ Flow cytometry
☒	☐ MRI-based neuroimaging

Plants

Seed stocks	n/a
Novel plant genotypes	n/a
Authentication	n/a
